# Noninvasive Tracking of Embryonic Cardiac Dynamics and Development with Volumetric Optoacoustic Spectroscopy

**DOI:** 10.1002/advs.202400089

**Published:** 2024-03-25

**Authors:** Maryam Hatami, Ali Özbek, Xosé Luís Deán‐Ben, Jessica Gutierrez, Alexander Schill, Daniel Razansky, Kirill V. Larin

**Affiliations:** ^1^ Department of Biomedical Engineering University of Houston Houston TX 77004 USA; ^2^ Institute for Biomedical Engineering and Institute of Pharmacology and Toxicology Faculty of Medicine University of Zurich Zurich 8057 Switzerland; ^3^ Institute for Biomedical Engineering Department of Information Technology and Electrical Engineering ETH Zurich Zurich 8092 Switzerland; ^4^ Department of Integrative Physiology Baylor College of Medicine Houston TX 77030 USA

**Keywords:** cardiovascular imaging, embryo development, functional imaging, heart, optoacoustic spectroscopy

## Abstract

Noninvasive monitoring of cardiac development can potentially prevent cardiac anomalies in adulthood. Mouse models provide unique opportunities to study cardiac development and disease in mammals. However, high‐resolution noninvasive functional analyses of murine embryonic cardiac models are challenging because of the small size and fast volumetric motion of the embryonic heart, which is deeply embedded inside the uterus. In this study, a real time volumetric optoacoustic spectroscopy (VOS) platform for whole‐heart visualization with high spatial (100 µm) and temporal (10 ms) resolutions is developed. Embryonic heart development on gestational days (GDs) 14.5–17.5 and quantify cardiac dynamics using time‐lapse‐4D image data of the heart is followed. Additionally, spectroscopic recordings enable the quantification of the blood oxygenation status in heart chambers in a label‐free and noninvasive manner. This technology introduces new possibilities for high‐resolution quantification of embryonic heart function at different gestational stages in mammalian models, offering an invaluable noninvasive method for developmental biology.

## Graphical abstract

This study describes the unique capability of a volumetric optoacoustic spectroscopy (VOS) platform for noninvasive assessment of anatomical and functional heart development in mice, as well as the quantification of blood oxygen saturation in the fetal heart. The research highlights the potential of VOS in understanding mammalian heart development and its application in diagnosing and preventing congenital heart disease.

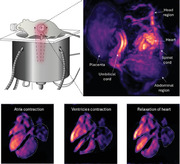

## 1 Introduction

Mammalian development relies on the proper formation and function of a four‐chambered embryonic heart.^[^ ^1^ ^]^ This complex process involves a sequence of changes that occur in a timely and coordinated manner, such as morphological remodeling or the formation of valves and connections.^[^ ^2^ ^]^ A detailed understanding of the interplay between these steps remains a long‐standing challenge in developmental biology. It is crucial to unravel the underlying causes of congenital heart disease (CHD), which is considered the most common birth defect and accounts for nearly one‐third of all major congenital anomalies.^[^ ^3^ ^]^ In its moderate form (e.g., ventricular hypoplasia), CHD may lead to a slight blending of placental blood in the embryonic right atrium, leading to severe illness shortly after birth. In its more severe form (e.g., hypoplastic left heart syndrome), CHD may result in total mixing of oxygenated and deoxygenated blood. Blood mixing reduces blood flow, as well as oxygen and nutrient supply to the brain, affecting the growth and development of other fetal organs.^[^ ^4^ ^]^ Therefore, a proper assessment of these and other CHD effects is of paramount importance.

An important part of our understanding of cardiogenesis stems from mouse‐embryo studies. Mouse models epitomize human cardiac development and disease, while being highly tractable for genetic manipulation.^[^ ^5^ ^]^ In particular, the assessment of murine embryonic cardiovascular structure and function is essential for investigating the etiology of CHD in humans.^[^ ^6^ ^]^ However, in vivo imaging and characterization of cardiac function in murine embryos are significantly challenged by their small size and in utero localization. Embryonic echocardiography has been extensively performed using high‐frequency ultrasound (US) systems.^[^ ^7^ ^]^ While it is challenging to obtain optimal imaging planes for cardiac assessments using standard 2D US,^[^ ^7a^ ^]^ the temporal resolution of 3D US is generally insufficient to accurately capture cardiac motion in mice. Optical imaging modalities, such as optical coherence tomography (OCT), have been employed for embryonic imaging with high spatiotemporal resolution,^[^ ^8^ ^]^ but lack the penetration depth to reach heart regions noninvasively. Longitudinal morphological imaging of embryonic development was performed using micro‐computed tomography (micro‐CT) and magnetic resonance imaging (MRI).^[^ ^9^ ^]^ However, the low temporal resolution of these full‐body tomographic approaches requires the use of self‐gating and co‐registration techniques for visualizing cardio‐dynamics,^[^ ^10^ ^]^ with the high cost and lack of molecular contrast being other key limitations.

Optoacoustic (OA) imaging enables deep‐tissue spectroscopic differentiation of distinctive tissue chromophores with unprecedented spatiotemporal resolution.^[^ ^11^ ^]^ This unique advantage has been exploited in important biological fields, such as oncology^[^ ^12^ ^]^ and neuroscience,^[^ ^13^ ^]^ and has been employed to render volumetric cardiac dynamics in murine models.^[^ ^14^ ^]^ This technique has also been used for longitudinal in utero morphological monitoring of embryonic development^[^ ^15^ ^]^ and placental oxygenation studies.^[^ ^16^ ^]^ In this study, we exploited the unique 5D (5D, spectroscopic real‐time volumetric) capacity of OA to comprehensively assess morphological, dynamic, and functional changes during mouse embryonic development, including multiparametric characterization of the fast‐beating embryonic cardiac cycle and blood oxygen saturation levels.

## 2 Results

### 2.1 In Vivo Imaging of Embryonic Development

**Figure 1:**
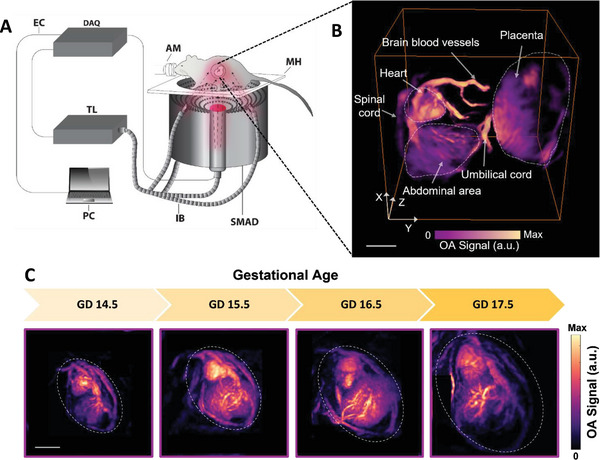
In vivo imaging of embryonic development. A) Schematic diagram of the VOS imaging system. TL: tunable laser; AM: anesthesia mask; MH: mouse holder; IB: illumination bundle; EC: ethernet cable; SMAD: spherical matrix array detector. B) 3D image of an embryo at gestational day (GD) 16.5 acquired with the VOS system at 800 nm wavelength. Maximum intensity projection (MIP) for an oblique view is presented. C) Developmental profile of murine embryos from GD 14.5 to GD 17.5. MIPs along the depth direction are shown. Scalebars = 2 mm.

The developed volumetric optoacoustic spectroscopy (VOS) imaging setup capitalizes on the large angular aperture of a spherical matrix array to provide high‐resolution accurate transabdominal volumetric images of murine embryos in vivo in a fully noninvasive manner (**Figure** **1**A; see Experimental Section for a detailed description). Reduced light attenuation at near‐infrared wavelengths (800 nm) facilitates reaching the depth of interest, while the effective field of view (FOV) is sufficient to cover the entire embryo and surrounding areas (Figure 1B). The real‐time imaging capability of the VOS further facilitated the identification of the embryonic beating heart, which was used as a beacon to properly position the embryos within the FOV during the acquisition of anatomical data across the peritoneal cavity of pregnant mice. Structural phenotyping of the volumetric scans highlighted different organs, such as the placenta and internal embryonic structures, including the abdominal region, brain vascular networks, and the spinal cord (Movies S1 and S2, Supporting Information). These are clearly visible, for example, in a representative maximum intensity projection (MIP) of the VOS image of the embryo at gestational day (GD) 16.5 (Figure 1B). Maturation and septation of the heart are the final developmental stages that occur after GD 12. The importance of this phase is manifested by the fact that most congenital heart malformations in humans are attributed to abnormalities in complex processes that involve the integration of growth and differentiation signals.^[^ ^17^ ^]^ However, the relatively large size of the heart prevents embryonic imaging with functional optical contrast at this developmental stage, even after intrauterine light delivery. The developmental profiles of embryos from GD 14.5 to GD 17.5, a critical gestational range for neurogenesis,^[^ ^18^ ^]^ could be accurately visualized with VOS, and the growth of embryonic anatomical organs over four consecutive gestational days could clearly be tracked (Figure 1C and Movie S3, Supporting Information). The VOS system was also capable of visualizing murine embryos at other gestational stages, for example GD 13.5 and GD 18.5, (Figure S1, Supporting Information), provided the resolution was sufficient to capture the structures of interest. Additionally, simultaneous imaging of multiple embryos within the same litter was successfully achieved in several experiments (Movie S4, Supporting Information).

### 2.2 Anatomical Imaging of the Embryonic Heart

**Figure 2:**
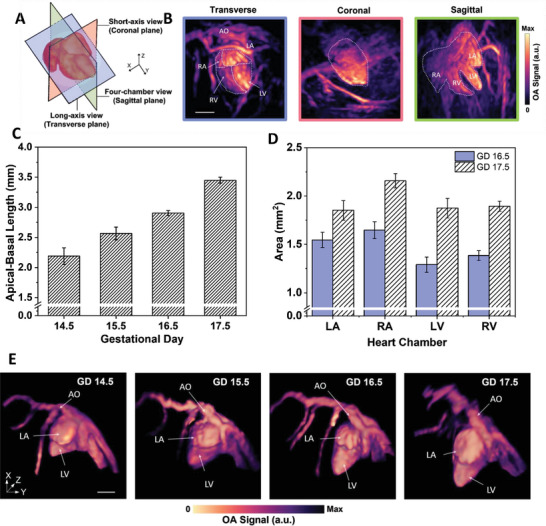
Anatomical developmental profile of the embryonic heart. A) Cardiac imaging planes corresponding to the long‐axis, short‐axis, and 4‐chamber views. B) Embryonic heart (GD 16.5) was visualized in the transverse, sagittal, and coronal planes. LA: left atrium; LV: left ventricle; RA: right atrium; RV: right ventricle; AO: aorta. C) Developmental embryonic heart (*n* = 5 per GD). Error bars represent standard errors across embryos of each GD. D) Variations in the area of the heart chambers within two gestational days (GD 16.5 and GD 17.5) (*n* = 5 per GD). LA: left atrium; LV: left ventricle; RA: right atrium; RV: right ventricle. Error bars represent standard errors across embryos of each GD. E) Noninvasive volumetric visualization of the embryonic heart showing stages of progressive heart development from GD 14.5 to GD 17.5. LA: left atrium; LV: left ventricle; RA: right atrium; RV: right ventricle; AO: aorta. Scalebars = 1 mm.

The high contrast and resolution of VOS images provide a powerful means of multiparametric anatomical characterization of the embryonic heart. The VOS imaging system developed in this study is based on the single‐shot excitation of the entire volume; that is, the effective integration time of the images is determined by a single nanosecond laser pulse. This avoids image blurring produced by other 3D imaging approaches because of the respiratory and cardiac motions of both the embryos and mother. It is also important to consider that limited‐view effects, which are common in OA imaging, are minimized by approximately 150° of angular coverage of the array.^[^ ^19^ ^]^ This resulted in an accurate representation of the blood‐filled heart chambers, facilitating quantification. Imaging planes corresponding to the long (transverse) axis, short (coronal) axis, and four‐chamber (sagittal) views of the embryonic heart were considered for the anatomical visualization and characterization of the heart (**Figure** **2**A). The heart volume can be segmented by considering the MIPs of the reconstructed images in these planes (Figure 2B and Movie S5, Supporting Information). Embryonic heart dimensions on different GDs were quantified using identical imaging planes. The heart size was measured in the parasternal long‐axis view. Specifically, the variation in the average apical‐basal length of the embryonic developmental heart from GD 14.5 to 17.5 was calculated (Figure 2C). Progressive proliferation of the developing heart is apparent in this plot. The embryonic heart undergoes a nearly 1.5‐fold increase in size from GD 14.5 to GD 17.5. Anatomical development was further analyzed by quantifying the embryonic heart chambers at two gestational stages, namely GD 16.5 and GD 17.5 (Figure 2D). A four‐chamber view of the heart, corresponding to the same cardiac phase in identical planes on both GDs, was used to segment and quantify the area of each heart chamber. Significant development of all the heart chambers was observed over a single GD. 3D views of the reconstructed, embryonic heart images for different GDs enabled anatomical phenotyping of the left ventricle, left atrium, and cardiac vasculature networks, highlighting the growth of each (Figure 2D). The anatomical development of the embryonic heart (Figure 2E) was quantified using this series of images.

### 2.3 Characterization of the Embryonic Cardiac Cycle

**Figure 3:**
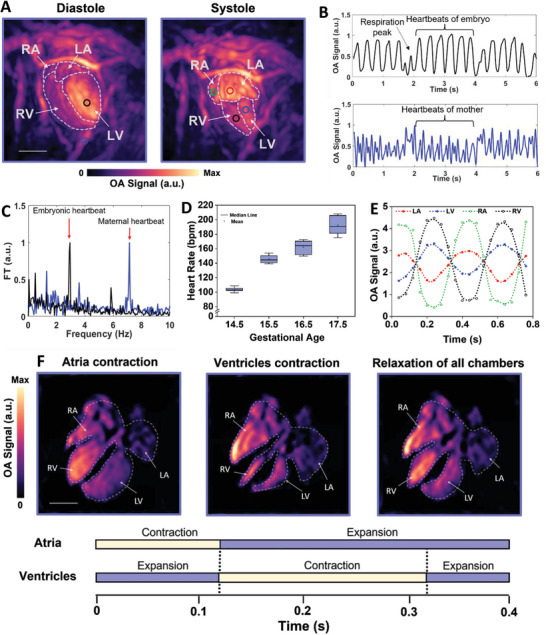
Characterization of embryonic cardiac cycle. A) Outlines of embryonic heart chambers (GD 16.5) for the systolic and diastolic phases of the heart on MIPs of volumetric images acquired at an illumination wavelength of 800 nm. Colored circles in the images correspond to the colored OA signal profiles. RA, right atrium; LA, left atrium; RV, right ventricle; LV, left ventricle B) examples of time‐lapse signal profiles for selected voxels marked by colored circles on the image of the heart in the diastolic phase. The signal from the black circle corresponds to the embryonic heart motion, whereas the signal detected from the blue circle corresponds to the mechanical motions of the maternal peripheral artery due to maternal heartbeats and respirations. C) Corresponding mean Fourier transform (FT) of signals from the embryonic heart (black curve) and maternal artery (blue curve). The peaks indicated by the red arrows represent embryonic and maternal heart rates. D) Boxplots of measured embryonic heart rate and embryonic developmental age (*n* = 5 per GD). Error bars represent standard errors across embryos of each GD. E) Temporal variations in signal intensity profiles in individual voxels in the four embryonic heart chambers (indicated by colored circles in the systolic phase image of panel a; left atrium: red, left ventricle: black, right atrium: green, and right ventricle: blue). Optical absorbance at 800 nm reflects the total blood volume. Temporal changes in signals acquired from image voxels in colored circles show changes in the blood volume in the corresponding region of the embryonic heart. F) Cross‐sectional images of the 4‐chamber embryonic heart (GD 16.5) at three different phases of the cardiac cycle, visualized at 800 nm. LA: left atrium; LV: left ventricle; RA: right atrium; RV: right ventricle; AO: aorta. Typical time intervals of cardiac phase events are illustrated in a bar graph. Scalebars = 1 mm.

Embryonic heart function was characterized by analyzing the cyclic contractions and relaxations of the heart chambers, considering the timing of the phases of the cardiac cycle (**Figure** **3**). The prominent anatomical features of the embryonic heart (right/left atria and right/left ventricles) in the systole and diastole phases were clearly identified in an image sequence of 400 frames of the embryonic heart and the cardiovascular network acquired at a repetition frequency of 25 Hz (Figure 3A). Contractions and expansions of embryonic heart chambers within a cardiac cycle can be characterized in the corresponding sequential frames of the embryonic heart (Figure S2 and Note S1, Supporting Information). Temporal variations in OA signals at selected points revealed the expected differences in embryonic and maternal heart rates (Figure 3B). The fundamental frequencies of the former, determined from a point in the left ventricle, and the latter, determined from a point in the maternal peripheral artery, were 2.95 Hz and 7.15 Hz, respectively (Figure 3C). The maternal respiration rhythm, with a fundamental frequency of 0.5 Hz, could also be identified in both profiles. The measured heart rate agrees well with the expected value for murine embryos at GD 16.5 subjected to isoflurane anesthesia.^[^ ^20^ ^]^ An expected progressive increase in embryonic heart rate with developmental stage was also observed (Figure 3D). This is arguably because of advances in the embryonic nervous system and the consequent increase in the functional capacity of the embryonic heart. The increased heart rate at late developmental stages was consistent with previously reported results obtained using high‐frequency US in pregnant CD‐1,^[^ ^7f^ ^]^ C57,^[^ ^7^, ^21^ ^]^ and Swiss/129 Sv mouse embryos.^[^ ^7g^ ^]^ Embryonic heartbeats were qualitatively perceived in the videos available in the online version of the journal (Movies S3 and S5, Supporting Information). Cardiac dynamics can be further evaluated quantitatively from the signals at selected points in each of the four heart chambers. Unlike in adulthood, signals from the two ventricles of the embryonic heart were in phase (Figure 3E). This is due to the fact that embryonic blood flow is derived from both ventricles operating in parallel, in opposition to the serial motion in the adult heart where blood flow is derived only from the left ventricle. Central shunting of the embryonic heart allows for these combined ventricular contractions and relaxations, which facilitate high cardiac output.^[^ ^22^ ^]^

The time difference between the peak signal values in the atria and ventricles over a complete cycle represents the embryonic heart rate. The time events of the cardiac cycle must be synchronized to maintain normal fetal cardiovascular function. Clinical studies have suggested that systolic time intervals of the fetal cardiac cycle can be used as indicators of cardiac function and fetal compromise.^[^ ^23^ ^]^ The time interval between cardiac phase events can be used to monitor the quality of the systolic and diastolic functions. Structural changes in the embryonic heart chambers within a cardiac cycle are highlighted in three cross‐sectional VOS images of the embryonic heart (GD 16.5) in the sagittal plane (Figure 3F). The corresponding timing of the embryonic cardiac phase is illustrated as a bar graph, representing the sequence of events within 400 ms of the embryonic cardiac cycle. As expected, the diastolic phase comprises nearly two‐thirds of the heart cycle.^[^ ^24^ ^]^ Ventricular systole occurs when the atria relax, and the ventricles contract to push blood out of the heart. This phase consisted of isovolumic contraction, ventricular ejection, and isovolumic relaxation, which took approximately 160 ms. During the last period of ventricular relaxation, atrial contraction (atrial systole) augments ventricular filling (ventricular diastole) before the next ventricular systole, a process that takes approximately 120 ms.

### 2.4 Blood Oxygen Saturation in Embryonic Heart Chambers

The 5D imaging capability of VOS provides unique opportunities for the functional evaluation of the embryonic heart. In this study, multispectral (multiwavelength) data acquisition was used to map blood oxygen saturation levels across the heart chambers and surrounding vasculature networks by capitalizing on the specific absorption spectra of hemoglobin in oxygenated (HbO_2_) and deoxygenated (Hb) states (Figure S3 and Note S2, Supporting Information).

**Figure 4:**
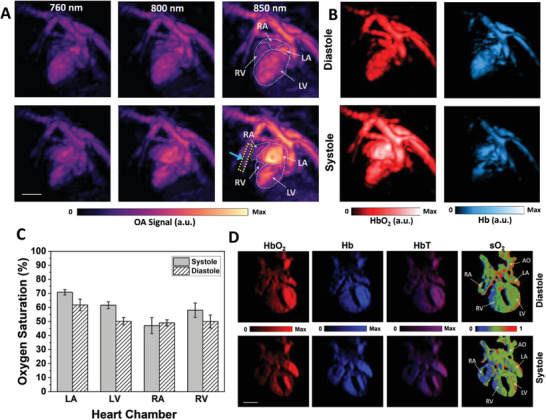
Functional information derived from embryonic cardiac images obtained using VOS. A) MIPs along the depth direction acquired at three different illumination wavelengths. Two phases of the embryonic heart cycle, systole and diastole, are displayed. Embryonic heart chambers are outlined on MIPs of images acquired at 850 nm for both the systolic and diastolic phases of the heart. The blue arrow marks the blood vessel carrying more oxygenated blood. B) Distribution of oxygenated (HbO_2_) and deoxygenated (Hb) hemoglobin in the embryonic heart during the two phases of the heart cycle corresponding to systole and diastole. C) Variations in the oxygen saturation state in embryonic heart chambers in the systolic and diastolic phases of the heart. Error bars represent standard deviations of oxygen saturations of various regions within each chamber of the heart. D) Maps of spectrally unmixed cross‐sectional VOS images of the four‐chambered embryonic heart. LA: left atrium; LV: left ventricle; RA: right atrium; RV: right ventricle; AO: aorta. Scalebars = 1 mm.

Images of the embryonic heart at GD 16.5, corresponding to optical excitation at 760, 800, and 850 nm, revealed a signal increase with wavelength, consistent with a high concentration of oxygenated hemoglobin (**Figure** **4**A). Images of the embryonic heart were analyzed during the quiescent phase of the respiratory cycle to avoid artifacts caused by breathing motion. This enabled the unmixing of HbO_2_ and Hb in different cardiac phases (Figure 4B). Considering that the extinction coefficients of both hemoglobin states are the same (isosbestic point) at a wavelength of approximately 800 nm,^[^ ^25^ ^]^ the VOS image acquired at this wavelength represents the overall biodistribution of hemoglobin. Multispectral imaging provides anatomical guidance on the embryonic vasculature. For example, a blood vessel carrying a high concentration of HbO_2_ was only observed at 850 nm (indicated by the blue arrow and dashed box in Figure 4A), which was further substantiated by spectrally unmixed images of HbO_2_ and Hb distributions (Figure 4B and Figure S4, Supporting Information). Variations in blood oxygen saturation have also been observed throughout the embryonic cardiac cycle. This suggests that the concentration of HbO_2_ is more substantial in the left atrium and aortic arch during the systolic phase, whereas a higher concentration of Hb is present in the heart chambers when the embryonic heart relaxes during the diastolic phase. Quantification of oxygen saturation in the embryonic heart chambers revealed oxygen‐rich blood in the left atrium during the systolic phase and oxygen‐poor blood in the right atrium during the diastolic phase (Figure 4C). This is similar to the expected mechanism of human fetal blood circulation.^[^ ^26^ ^]^ In the human fetus, oxygenated blood in the left atrium mixes with pulmonary blood and then passes into the left ventricle, providing oxygenated blood (approximately 65% saturation) to the coronary arteries and fetal brain. Less oxygenated blood (approximately 55% saturation) fills the right heart and enters the lungs through the right ventricle.^[^ ^26^ ^]^ The spectrally unmixed HbO_2_, Hb, and total hemoglobin biodistributions, along with the oxygen saturation map over two selected cross‐sections of the VOS images at the systolic and diastolic phases of the embryonic four‐chambered heart, were consistent with these values (Figure 4D). It has been previously reported that HbO_2_ is the dominant component in all chambers of the adult murine heart, with Hb detected only in the right ventricle.^[^ ^14^, ^27^ ^]^ However, the embryo has lower oxygen consumption owing to its low metabolic rate, limited respiratory effort, and thermoregulation provided by the mother. Therefore, an environment with a relatively low oxygen content is needed to protect the embryo against oxidative stress, and the embryo thrives in a hypoxic environment. The umbilical blood is 70–80% saturated, and the oxygen saturation in the fetal heart is approximately 50–65%.^[^ ^28^ ^]^ The unmixed VOS images showed that HbO_2_ was the prevalent component in the embryonic cardiac chambers, although both HbO_2_ and Hb forms existed in the heart chambers and cardiac vascular network, which is consistent with the expected difference in oxygen saturation in the embryonic heart compared to that in adults.

## 3 Discussion

The focus of this study was to exploit the unique capabilities of a truly noninvasive high‐resolution deep‐tissue VOS imaging approach to visualize the morphology of murine embryos in vivo and dynamically assess fetal cardiac function. The results obtained demonstrate that the high spatiotemporal resolution provided by the system can overcome the challenges associated with small embryonic size and the presence of a muscular uterus, and that the valuable optical‐absorption‐based contrast further enables the functional characterization of hemodynamics and embryonic anatomical structures. To monitor the evolution of embryonic heart function during the subsequent developmental stages, we performed VOS of the abdominal region of pregnant CD‐1 mice on GDs 14.5 to 17.5. Monitoring the development of a single embryo over successive gestational days, however, can be challenging and requires a larger field of view, which can be achieved by, for example, rotating or translating the US transducer array to ensure accurate image registration. In several experiments, images of multiple embryos within the same litter were captured simultaneously. However, concurrent imaging of multiple embryos presented challenges due to the limited field of view, particularly when imaging embryos at later gestational stages. These challenges were further exacerbated when the embryos were obstructed by maternal internal organs, such as the intestines, or when the orientation of some embryos hindered capturing a proper view of the heart.

Images at an 800 nm wavelength facilitated the identification of embryonic organs and the placenta within the maternal abdominal cavity. Embryonic heartbeat pulsations were captured at a sufficiently high frame rate (25 Hz) to distinguish the temporal profiles of the changes in different heart chambers from those of the mother, thus providing information on embryonic heart ventricular function. Specifically, it was possible to observe both ventricles contracting and relaxing simultaneously, as opposed to sequential motion in the adult heart. Alterations in heart rate are well‐documented in mice subjected to pharmacological stimulation and mouse models of chronic heart failure.^[^ ^29^ ^]^ The methodology introduced in this study holds great promise for assessing the severity of congenital abnormalities and the efficacy of therapeutic interventions. Limited‐view artifacts associated with insufficient angular coverage in OA imaging have been reduced by utilizing spherical arrays, which provide accurate volumetric images of vascular networks.^[^ ^30^ ^]^ However, the large inter‐element pitch of these arrays hampers US beamforming for pulse‐echo US imaging, typically achieved with linear arrays with densely‐packed elements. Recently, a new hybrid spherical array has been proposed incorporating a central segment of cylindrically‐focused elements to combine volumetric OA and cross‐sectional US data acquisitions.^[^ ^31^ ^]^ This innovation could potentially be used to provide additional anatomic information on the embryo and placenta. VOS is a particularly versatile and powerful functional imaging method that employs optical excitation at multiple wavelengths.^[^ ^32^ ^]^ Imaging at wavelengths of 760, 800, and 850 nm enabled quantification of embryonic functional parameters, such as HbO_2_, Hb, and total hemoglobin in the embryonic chambers and structures. The measured oxygen saturation in the heart chambers revealed the expected presence of mixed venous oxygenation in the embryo, which caused significant oxygenation differences in the chambers compared to the adult heart. Analysis of oxygenation maps over different embryonic heart phases (systole and diastole) has provided new insights into embryonic blood circulation. The embryonic heart primarily serves to circulate blood throughout the developing organs, while oxygenation mainly occurs through the placenta.^[^ ^28^ ^]^ However, the oxygen saturation of the blood changes during the cardiac cycle. During systole, the heart contracts and pumps a mixture of oxygenated and deoxygenated blood into the circulation. This mixing occurs because of unique structures in the fetal circulation, such as the foramen ovale and ductus arteriosus, which allow blood from the right and left sides of the heart to mix. During diastole, the heart relaxes, and the chambers fill with blood that is a mixture of oxygenated blood from the placenta and deoxygenated blood from the fetal body. The oxygen saturation in the embryonic heart and its fluctuations within the cardiac cycle become more pronounced in cases of impaired heart function. For instance, in mouse models of congenital heart disease, structural heart defects can lead to the mixing of blood between ventricles, resulting in oxygen saturation levels that differ from those in a healthy heart. Alterations in fetal heart oxygenation would also be significant in cases of prenatal exposure to substances that affect the embryonic heart rate and placental oxygenation.

The heart is the first essential organ to develop during organogenesis and is the central organ in fetal adaptive mechanisms in the face of various perinatal complications. Fetal cardiac impairment can be caused by a secondary adaptive mechanism or intrinsic myocardial disease. Impaired fetal heart function correlates with functional cardiac anomalies and heart defects in adulthood. The complex process of heart development involves the transformation of a primitive tubular peristaltic pump into a more complex organ featuring different chambers capable of regulating both the pulmonary and systemic circulation. Heart development has been extensively studied in wild‐type and mutant mouse models with a cardiovascular system structurally more similar to that of humans than in other species used in biomedical research.^[^ ^20^, ^33^ ^]^ Mice provide an opportunity to examine normal heart development and alterations in heart morphogenesis and function.^[^ ^34^ ^]^ Functional imaging of murine embryonic cardiac models can contribute to the understanding of heart development in mammals. A better understanding of the underlying genetic programs driving the growth and differentiation of organs is a longstanding challenge for developmental biologists and is key to unraveling the environmental and genetic factors that may lead to CHD.^[^ ^35^ ^]^

During the critical window of organ development, the supply of nutrients and oxygen is essential for meeting fetal metabolic demands. Optimal maternal health facilitates normal embryogenesis and fetal growth, while ensuring fetal survival. However, in many situations, including pre‐existing maternal anemia and chronic inflammation, the fetus can be exposed to stress during intrauterine life. Under these circumstances, maternal physiology is altered, and unfavorable intrauterine conditions are created, which can result in maldevelopment and defective fetal growth. Intrauterine growth restriction (IUGR), which is mainly caused by uteroplacental vascular insufficiency, occurs in up to 10% of pregnancies.^[^ ^36^ ^]^ IUGR induces the redistribution of fetal cardiac output to preserve oxygen and nutrient supply to essential fetal organs, such as the brain and heart.^[^ ^37^ ^]^ Studies have shown an association between IUGR and changes in cardiac systolic and diastolic functions.^[^ ^38^ ^]^ These cardiac function changes appear to persist into adulthood.^[^ ^39^ ^]^ CHD can also affect the growth of other organs during fetal development. Possible structural and anatomical changes in the heart are indicative of CHD. Fetal heart function has been analyzed by quantifying cyclic contractions and relaxations of the heart chambers, timing of phases of the cardiac cycle, and heart rate.^[^ ^24^ ^]^ Functional evaluation of the embryonic heart also provides significant information regarding the hemodynamic state of the heart and fetal cardiac adaptation to intrauterine changes. New functional and dynamic imaging approaches capable of shedding light on the underlying causes of abnormal fetal development in mice can facilitate better strategies for the prevention and treatment of CHD. CHD manifests as various defects in the structure of the heart or great vessels, which can be potentially quantified using the developed VOS platform. The resolution of the system was demonstrated to enable differentiation of internal structures in the embryonic heart for GDs 14.5 to 17.5. Consequently, ventricular septal defects, such as openings between the two ventricles, are expected to be detectable at these stages. Additionally, such defects can result in mixing of blood from both ventricles, leading to different oxygen saturation levels compared to a healthy heart. These changes can be detected by leveraging the multispectral imaging capabilities of the system. Furthermore, the temporal resolution of the system is sufficient to accurately characterize the murine cardiac cycle even at 600 beats per min. Therefore, various types of arrhythmias associated with CHD are expected to be detected.

Human development, similar to that of mice and other mammals, critically relies on proper formation of the embryonic heart. Up to 10% of miscarriages are ascribed to perturbations in heart development or function.^[^ ^36^ ^]^ Heart abnormalities are diagnosed in approximately 1% of newborns, and their management represents an unmet clinical need.^[^ ^3b^ ^]^ Assessment of fetal cardiac functional characteristics during embryonic development is instrumental in the diagnosis and prevention of future pathological cases in adulthood. This consists of an evaluation of the changes in the anatomical structure and functional parameters of the heart during fetal development. Cardiac dynamics are also evaluated to assess the quality of contraction and relaxation of myocardial fibers, their ability to preserve fetal blood circulation, and the fetal heart rate. Early identification of cardiovascular abnormalities and specific interventional measures in cases of gestational pathologies is of major clinical significance. During the fetal cardiac evaluation, sequential multiparametric analysis of the embryonic heart is performed.^[^ ^24^, ^39^ ^]^ The unique advantages of multispectral OA tomography have recently been exploited in initial clinical trials to foster clinical translation of this technology.^[^ ^40^ ^]^ Hence, fetal imaging in humans can be performed using this imaging technique. However, it is important to consider that the reachable depth from the accessible surface is limited to a few centimeters; thus, optimal imaging performance may be achieved via in utero light delivery. The multiparametric volumetric spectroscopic characterization performed in this study could potentially be performed on human fetuses. This can significantly enhance the information provided by fetal cardiac evaluation procedures and facilitate clinical decision making.

## 4 Conclusion

VOS provides otherwise unattainable anatomical, dynamic, and functional information about murine embryos. High‐resolution volumetric images of the internal embryonic structures were rendered in real‐time, which enabled a comprehensive analysis of the periodic motion of the heart. Analysis of images acquired at multiple excitation wavelengths further exploits the unique absorption signature of blood components to resolve the biodistribution of oxygenated and deoxygenated hemoglobin and quantify oxygen saturation in different heart chambers. The fully noninvasive nature of VOS is essential for longitudinal studies to provide new insights into the dynamic progression of CHD in mutant embryos. These unique advantages greatly impact cardiovascular research and can potentially be used for fetal cardiac evaluation in humans if the imaging system is optimized for this purpose.

## 5 Experimental Section

### Volumetric Optoacoustic Spectroscopy Setup

The newly developed VOS setup comprises a multi‐angle illumination fiber bundle, US detection array, data acquisition system, and a personal computer (PC) for image reconstruction and data analysis. An integrated Nd: YAG pumped, wavelength‐tunable optical parametric oscillator (OPO) laser source (Spitlight DPSS OPO, InnoLas Laser GmbH, Germany) capable of generating pulses with an adjustable pulse repetition frequency (PRF) of up to 100 Hz and a pulse duration of <10 ns was used for OA signal excitation. The wavelength of the laser was tuned from 650 to 1300 nm on a per‐pulse basis. Imaging was performed at a single wavelength of 800 nm for anatomical identification and labeling of major embryonic organs. On the other hand, multispectral (multiwavelength) imaging was performed for estimation of oxygen saturation.

Laser‐induced US emission from the tissue was detected using a US transducer matrix array (Imasonic Sas, France) with a central frequency of 7 MHz and a −6 dB detection bandwidth of 60%. The effective FOV provided by the spherical transducer array was approximately 15 ×  15 ×  15 mm^3^, allowing a relatively isotropic resolution of approximately 114 µm (Figure S4 and Note S3, Supporting Information) around the center of the sphere. The US transducer consisted of a 2D array of 512 trapezoidal piezocomposite elements, each with an approximate elementary area of 12 mm^2^, distributed over an active spherical surface covering an angular aperture of 150°. A central aperture of 8 mm in diameter and three equally spaced lateral apertures, each 4 mm in diameter and oriented at 45°, were used for optical‐fiber‐guided light delivery. The spherical array was positioned facing upward, fixed using an O‐ring holder, and sealed at the edges before being filled with an agar solution. An agar matrix and an acoustic gel were used to provide a coupling medium for efficient US transmission. The laser beam was directed toward the imaging object via a four‐arm optical fiber bundle (CeramOptec GmbH, Germany). Each arm of the bundle is coupled to the apertures of the US matrix array, thus providing illumination to the object in both axial and azimuthal directions. The laser source was operated at a PRF of 25 Hz for image acquisition at 800 nm, with a pulsed laser fluence of 15 mJ cm^−2^.A parallel digital acquisition (DAQ) platform (Falkenstein Mikrosysteme GmbH, Germany) triggered by the output of the laser was used to digitize the signals detected from the 512 transducer channels at 40 MS s^−1^, which enabled real‐time volumetric visualization. The sampled signal data were transmitted over a 1 GB Ethernet connection to a PC. The data were then processed in real‐time using a computer accelerated by a graphical processing unit (GPU).

### Image Reconstruction

All reconstruction and processing steps were performed using MATLAB (R2021b, MathWorks, Inc., MA, USA). The signals were first deconvolved with the impulse response of the US elements and then bandpass‐filtered with cut‐off frequencies 0.1 MHz and 9 MHz. The images were then reconstructed using a GPU‐implemented back‐projection reconstruction algorithm.^[^ ^41^ ^]^ Specifically, 3D images were reconstructed for a FOV of 10 × 10 × 10 mm^3^ (256 × 256 × 256 voxels). All raw 4D (3D+time) VOS data were stored for offline image reconstruction and postprocessing.

### Spectroscopic Unmixing

The oxygenation state of the embryonic heart indicates the embryonic cardiac performance during blood extraction and fetal blood circulation. Multispectral VOS imaging was performed at multiple wavelengths to obtain information regarding the state of blood oxygenation in the embryonic heart. Specifically, wavelengths of 760, 800, and 850 nm were used to match the absorption peaks of deoxygenated hemoglobin (Hb), its isosbestic point, and the high absorption of oxygenated hemoglobin (HbO_2_). 5D VOS data were acquired at a PRF of 50 Hz for further analysis. Linear regression spectral unmixing was used to generate biodistribution maps of HbO_2_ and Hb, based on the reference absorption spectrum of hemoglobin.^[^ ^25^ ^]^ The signal intensity depends on both the optical absorption coefficient of the optically absorbing constituents and the distribution of light fluence within the tissue. Melanin is another major optical absorber in tissue, and the skin color of mice could influence the quantification of oxygen saturation. A higher concentration of melanin in darker skin tones produces a stronger OA signal at the skin surface, which affects the penetration depth of the signal and the accuracy of oxygen saturation measurements.^[^ ^42^ ^]^ However, for the purposes of this study, skin absorption was assumed to be constant across all the imaged embryos. The dependence of light fluence on the optical wavelength induces spectral distortion (i.e., spectral coloring) in deep‐tissue locations. In this study, the light fluence distribution over the embryonic cardiovascular region was assumed constant.

### Animal Preparation and In Vivo Imaging

Timed mating of the CD‐1 male and female mice (Envigo RMS LLC, IN, USA) was performed. Pregnancy was confirmed on the following day by observing the seminal plug after overnight mating. In staging the embryos, gestational day (GD) 0.5 was defined as noon on the day the vaginal plug was observed. All experimental procedures, including animal handling, were performed in compliance with the guidelines of the University of Houston Institutional Animal Care and Use Committee. Prior to in vivo imaging, pregnant mice were anesthetized by inhalation of 2.5% isoflurane (Aerrane, Baxter Healthcare, Deerfield, IL, USA) in 1 L min^−1^ oxygen (O_2_) in an induction chamber for 5 min. After induction of isoflurane anesthesia, the mouse was placed in a supine position on a heating pad, and the abdominal fur was removed using depilatory cream to improve contact with the agar matrix. The depilatory cream was removed 1 min after application, using wet and dry gauze to prevent skin damage. Anesthesia was maintained during the imaging session by inhalation of a mixture of 1‐2% isoflurane in 1 L min^−1^ O_2_ through a nose cone attached to an open‐circuit isoflurane vaporizer. For imaging, the mice were placed in a prone position over an agar‐filled US transducer array. A pulse oximeter was attached to the rear limb of the animal to provide real‐time monitoring of the heart rate and systemic arterial oxygen saturation. Body temperature was monitored with a rectal probe and maintained at $36\pm 0.5^{\circ }C$ C using a heating lamp placed above the mouse to minimize physiological alterations to the maternal and embryonic cardiovascular systems due to anesthesia. Pregnant mice were imaged on GDs 14.5–17.5 (five mice per GD). The signals were acquired at 25 Hz PRF over a sequence of 400 frames to capture the cardiac cycle dynamics. Signal averaging was not performed. Considering that the reported heart rate of mouse embryos is approximately 2–3 Hz,^[^ ^7^, ^21^ ^]^ a volumetric frame rate of 25 Hz provided approximately ten volumetric images during each cardiac cycle, which is sufficient for heart function analysis.

### Statistical Analysis

The parasternal long‐axis plane of the embryonic heart was considered for the analysis and quantification of embryonic cardiac parameters, such as heart rate and heart size. The size of the embryonic heart was measured from the base to the apex to quantify the heart development. Images of the hearts of different GDs were chosen from the same anatomical plane to ensure consistency in size measurements. All quantification results are presented as the mean ± SEM for each group.

The temporal profiles of signal intensities at different points in the VOS images of the heart were analyzed to render and visualize cardiac dynamics and retrieve embryonic anatomical and cardiac hemodynamic data. The heart rate is the main determinant of embryonic cardiac output. Cardiac dynamics were quantified using the time profiles extracted from different voxels of the heart. The Fourier transform (FT) of the time profiles was calculated to quantify the frequency of the signals on each GD, from which the embryonic heart rate was estimated.

The time intervals of the systolic and diastolic phases and the order of these events were characterized using the temporal profiles of signals from the embryonic heart chambers. Based on the structural changes in the heart chambers and the temporal evolution of signals from the heart, different steps of the diastolic and systolic phases were identified, and the duration of each step was quantified.

## Conflict of Interest

The authors declare no conflict of interest.

## Supporting information

Supporting Information

Supplemental Movie 1

Supplemental Movie 2

Supplemental Movie 3

Supplemental Movie 4

Supplemental Movie 5

## Data Availability

The data that support the findings of this study are available in the supplementary material of this article. Additional supporting data from the findings of this study are available from the corresponding author upon reasonable request.
