# Supplementary material for: Noninvasive Tracking of Embryonic Cardiac Dynamics and Development with Volumetric Optoacoustic Spectroscopy
Source: Adv Sci (Weinh). 2024 Mar 25;11(22):2400089. doi: 10.1002/advs.202400089 (PMC11165471; doi:10.1002/advs.202400089)
Supplement: Supplementary file 1 — Supporting Information [file ADVS-11-2400089-s006.pdf]

ADVANCED SCIENCE
Open Access

Supporting Information

for *Adv. Sci.*, DOI 10.1002/advs.202400089

Noninvasive Tracking of Embryonic Cardiac Dynamics and Development with Volumetric Optoacoustic Spectroscopy

Maryam Hatami, Ali Özbek, Xosé Luís Deán-Ben, Jessica Gutierrez, Alexander Schill, Daniel Razansky and Kirill V. Larin**

Supporting Information

Noninvasive Tracking of Embryonic Cardiac Dynamics and Development with Volumetric Optoacoustic Spectroscopy

Maryam Hatami, Ali Özbek, Xosé Luís Deán-Ben, Jessica Gutierrez, Alexander Schill, Daniel Razansky *, *and Kirill V. Larin* *

This document includes:

Notes S1 to S3
Figures S1 to S5
Legends for movies S1 to S5

Other Supplementary Material for this manuscript includes the following:

Movies S1 to S5

Supplementary Text

All experimental procedures, including animal handling, were performed in compliance with the guidelines of the University of Houston Institutional Animal Care and Use Committee, protocol No. 15037.

Note S1: Evaluation of embryonic heart cycle

To evaluate the embryonic cardiac cycle, we analyzed optoacoustic (OA) images of the embryonic heart across various stages at gestational day (GD) 16.5 (Figure S2). Captured at a frequency of 25 Hz, these images sequentially depict the heart's motion throughout the cycle, showcasing distinct morphological changes and phases of systole and diastole. The initial frame captures the ventricular wall at the end of systole, while the subsequent frame illustrates the onset of ventricular expansion and the isovolumetric relaxation (ventricular diastole). This expansion continues for 40 ms, with a later frame displaying the complete relaxation of all heart chambers. As the ventricles further expand, the pressure in the ventricles starts to decline. Concurrently, the initiation of atrial contraction propels blood towards the relaxed ventricles, a process vividly depicted in frame 5. The sequence advances to the next frame, depicting the heart in diastole. After 40 ms, by frame 7, atrial expansion starts with no change in ventricular volume. Frame 8 then illustrates the ventricular walls beginning to contract, making the start of isovolumetric contraction. This phase precedes the ejection of blood during systole captured in the concluding frames 9 to 12.

Note S2: Embryonic oxygen saturation state

The OA image captured at an 850 nm illumination wavelength reveals higher signal intensity than those obtained at 760 nm and 800 nm, as depicted in Figure S3 A. This enhancement in signal strength from organs with oxygen-rich blood is attributed to the increasing optical absorption (extinction coefficient, ε) of oxygenated blood with wavelength (λ), as shown in Figure S3 B. The OA image at 800 nm, where oxygenated (HbO_2) and deoxygenated (Hb) hemoglobin absorptions converge (isosbestic point), displays total hemoglobin absorption

contrast. Spectrally unmixed biodistribution maps of the HbO_2 and Hb, presented in Figure S4 A and B, highlight a pronounced HbO_2 signal from the embryonic cardiovascular system and the umbilical vessels. Molecular-specific volumetric optoacoustic spectroscopy (VOS) enables the identification of embryonic vasculature. Notably, the arrow in Figure S4 A points out the vessel with deoxygenated blood, which is visible on the Hb map but not on the HbO_2 map. Conversely, arrows in Figure S4 B indicate vessels with oxygenated blood, absent from the Hb map. The overlay of these images, as illustrated in Figure S4 C, features both oxygenated and deoxygenated blood vessels and organs, providing a comprehensive view of the embryonic vasculature.

Note S3: Spatial resolution characterization

An optically transparent agar phantom embedded with black paramagnetic polyethylene microspheres (Cospheric LLC Santa Barbara, CA, size 38–45 µm) was utilized to characterize the VOS system capability. The laser wavelength was set to 680 nm with a peak energy output of 13 mJ. The agar phantom was made using 1.3% agar powder (05038; Sigma-Aldrich, St. Louis, MO) dissolved in distilled water. Next, black microspheres were sparsely embedded in this tissue-mimicking agar matrix. To evaluate the resolution of the system, the agar phantom was moved against the ultrasound transducer array in various directions from the center of the spherical transducer array.

The spatial resolution at each position was evaluated by determining the full width at half maximum (FWHM) of the reconstructed image profiles of the microspheres near the center of the ultrasound detection array. First, the point spread function (PSF) was calculated using the three-dimensional (3D) back-projection algorithm. Then, the Gaussian functions representing the signal profile of the absorbing microsphere were convolved with the PSF. The FWHM of the resulting signal was quantified, and the average squared difference between the measured FWHM and the diameter of the microsphere was considered the resolution. The field of view was defined as the volume where the amplitude of the reconstructed microspheres at a certain

position relative to the transducer is greater than 50% of the maximum amplitude achieved at the center of the field of view. An approximately isotropic volume of 15×15×15 mm^3 was measured as the effective field of view. A 3D reconstructed image of a microsphere at the geometrical center of the transducer array is displayed in Figure S5 A. Figure S5 B shows the maximum intensity projections of the OA signals along the laser direction (Cross-section perpendicular to the Z-axis) and in two perpendicular directions on the normal plane to the ultrasound transducer axis (Cross-sections perpendicular to the X and Y axes). The variations in one-dimensional OA signal profiles along each dashed line in Figure S5 A are plotted in Figure S5 C. Figure S5 D illustrates the reconstructed size of the microsphere at various distances from the center of the ultrasound array. The corresponding PSF was determined for each location using the back-projection algorithm within a $10 \times 10 \times 10$ mm^3 volume, comprising $400 \times 400 \times 400$ image voxels. This figure highlights a relatively isotropic resolution of the system, with enhanced resolution near the center, which slightly degrades away from the center. By deconvolving the final size of the microsphere from the measured FWHM of the Gaussian fitted curve, the effective axial and lateral resolutions were found to be approximately 107 μm and 114 μm, respectively, within 1 mm^3 volume centered on the focus of the spherical transducer array.

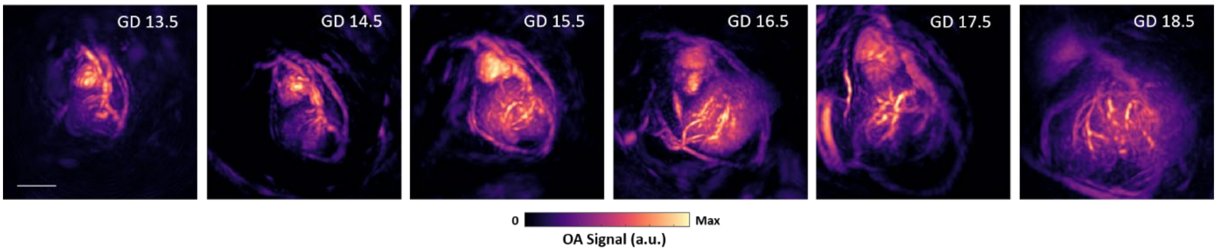

Figure S1. Outline of embryonic development from GD 13.5 to GD 18.5. Volumetric OA images acquired at an 800 nm illumination wavelength. Scalebar = 2 mm.

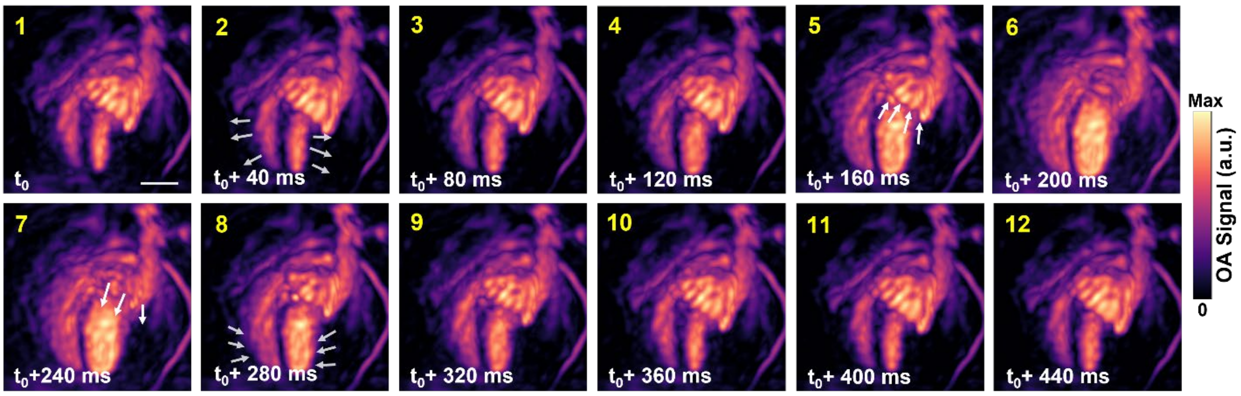

Figure S2. Embryonic cardiac dynamics (GD 16.5) visualized at an 800 nm illumination wavelength. OA images of the heart are shown for twelve timeframes (1–12). Arrows indicate the directions of expansion and contractions of the heart chambers. Scalebar = 2 mm.

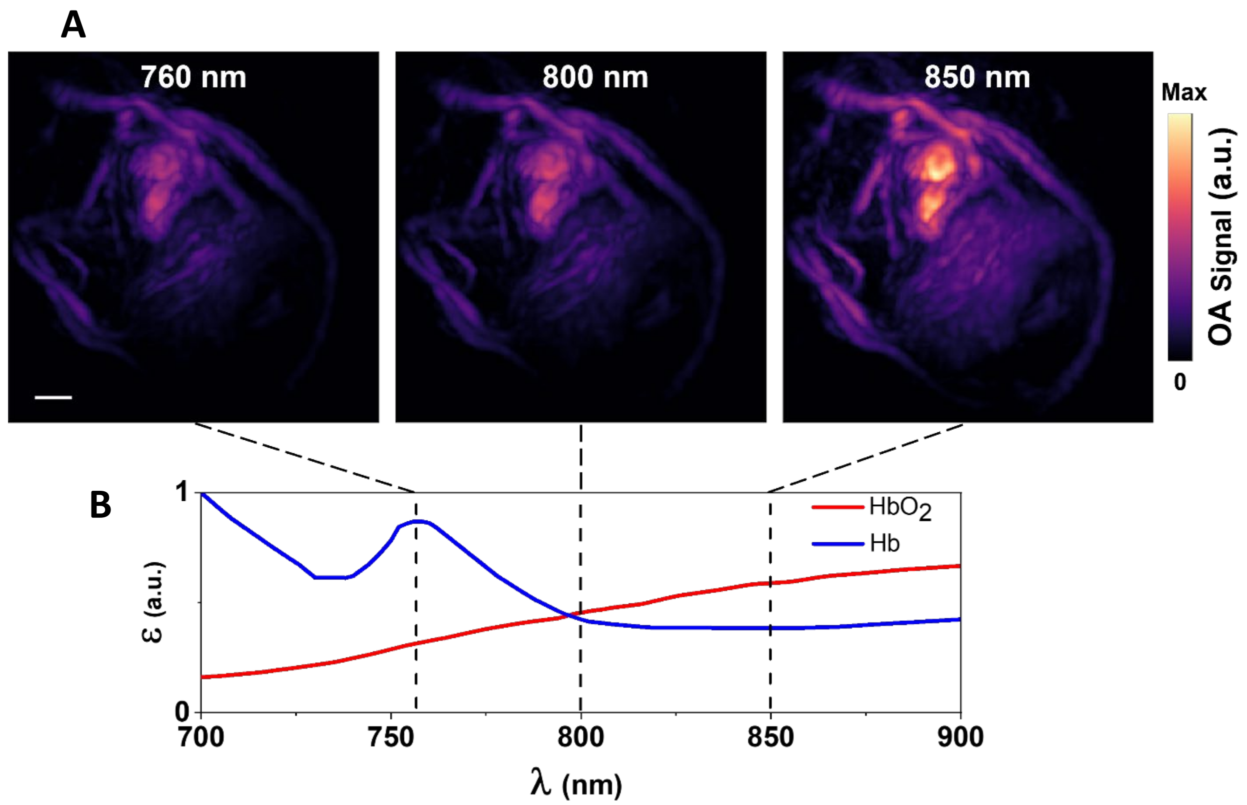

Figure S3. Multispectral VOS images of the embryo (GD. 16.5) in the transverse plane. (**A**) VOS images acquired at three different excitation wavelengths. Scalebar = 1 mm. (**B**) Variations in the normalized extinction coefficient ($\boldsymbol{\varepsilon}$) of Hb and HbO_2 with the wavelength ($\boldsymbol{\lambda}$).

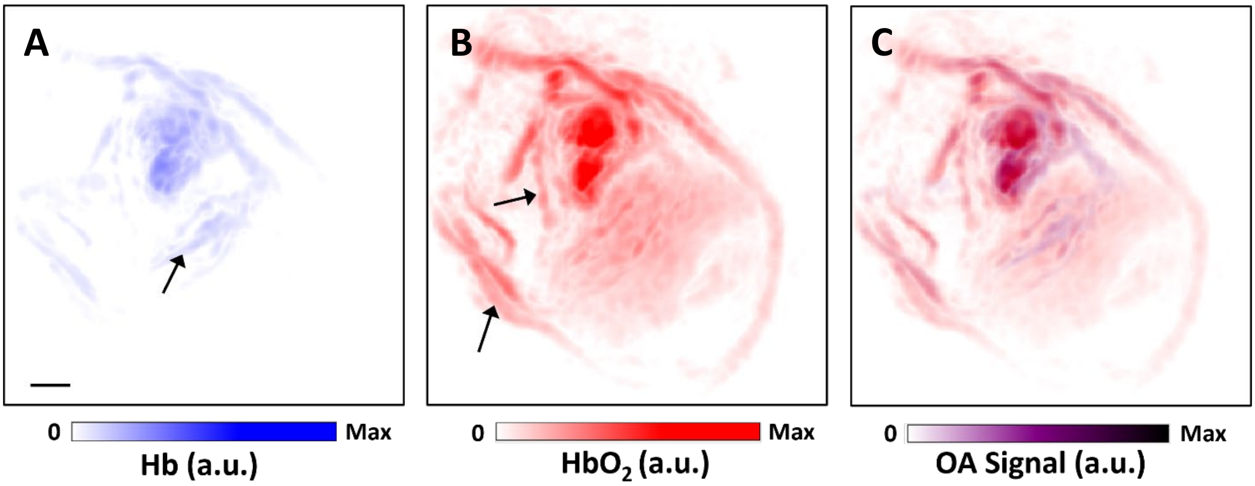

Figure S4. Spectrally unmixed VOS images of the embryo (GD. 16.5) in a transverse plane. (**A**) Map of the unmixed distribution of Hb. (**B**) Corresponding map HbO_2. (**C**) Overlay of spectrally unmixed VOS images of Hb and HbO_2. Uniform threshold limits were employed on all three images for comparison. Scalebar = 1 mm.

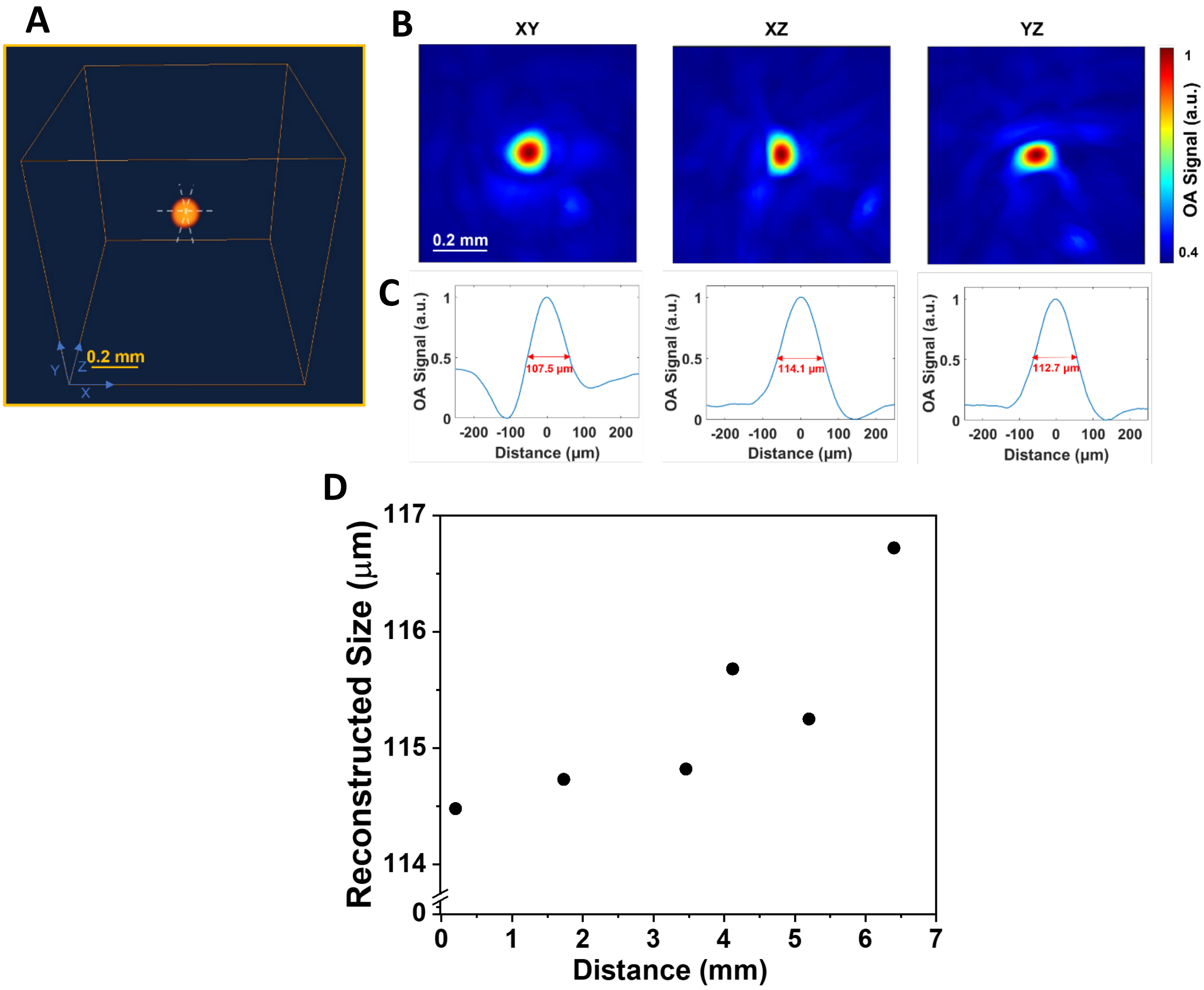

Figure S5. Experimental characterization of spatial resolution of the VOS system. (**A**) Volumetric image of a 40 μm diameter microsphere positioned at the center of the spherical array. (**B**) Maximum intensity projections of the VOS signals along the laser direction (Z) and two lateral directions (X and Y) normal to the laser direction. (**C**) Corresponding VOS signal profiles along the white dashed lines in A. (**D**) Size of the reconstructed microsphere as a function of the distance from the geometrical center of the transducer array.

Supplementary Movies

Movie S1 (MP4). Rotating view of the volumetric image of an embryo with a beating heart at GD 16.5. Motions of all four heart chambers are featured, along with the structural visualization of various organs, such as the placenta and umbilical cord, in addition to internal embryonic structures, including the abdominal region, brain vascular networks, and the spinal cord. The volumetric data for this visualization were captured using an 800 nm illumination wavelength at a frame rate of 25 Hz.

Movie S2 (MP4). Structural phenotyping overview of an embryo's entire body at GD 16.5. The flythrough video begins with a detailed view of the embryonic brain vasculatures, then transitions to show the volumetric view of the beating heart, followed by the abdominal region below the heart, and concludes with the umbilical cord. The data for this visualization were acquired at an 800 nm wavelength and a frame rate of 25 Hz.

Movie S3 (MP4). Functional and anatomical development of embryonic organs over four consecutive gestational days. Progressive increase of the embryonic heart rate and the structural heart development as gestation advances are highlighted. The volumetric data were acquired at the 25 Hz rate and a wavelength of 800 nm. The field of view (FOV) used for visualizing the embryos at all GDs is $10\times10\times10$ mm^3.

Movie S4 (MP4). Functional and anatomical visualization of two embryos (GD 16.5) from the same litter. The placenta, abdominal regions, hearts, and spinal regions of two embryos are featured, along with the cardiac dynamics of both embryos. The volumetric data were acquired at a frame rate of 25 Hz and an illumination wavelength of 800 nm.

Movie S5 (MP4). Various projection views of the embryonic beating heart at GD 16.5. The embryonic heart is visualized across three cardiac planes: transverse (long-axis view), coronal (short-axis view), and sagittal (four-chamber view), using an illumination wavelength of 800 nm and a frame rate of 25 Hz. The transverse view helps identify the embryonic heart and cardiovascular system. The sagittal section displays both ventricles, atria, and the interventricular septum, offering an optimal view for examining changes in cardiac ventricular dimensions to assess ventricular diastolic function. The sagittal plane effectively captures the simultaneous contractions and expansions of the ventricles.
